# Regulation of Connexin-Based Channels by Fatty Acids

**DOI:** 10.3389/fphys.2017.00011

**Published:** 2017-01-24

**Authors:** Carlos Puebla, Mauricio A. Retamal, Rodrigo Acuña, Juan C. Sáez

**Affiliations:** ^1^Instituto de Ciencias Biomédicas, Facultad de Ciencias de la Salud, Universidad Autónoma de ChileSantiago, Chile; ^2^Centro de Fisiología Celular e Integrativa, Facultad de Medicina, Clínica Alemana Universidad del DesarrolloSantiago, Chile; ^3^Departamento de Fisiología, Facultad de Ciencias Biológicas, Pontificia Universidad Católica de ChileSantiago, Chile; ^4^Centro Interdisciplinario de Neurociencias de Valparaíso, Intituto Milenio, Universidad de ValparaísoValparaíso, Chile

**Keywords:** gap junction channel, hemichannel, connexon, pannexon, G-protein coupled receptor

## Abstract

In this mini-review, we briefly summarize the current knowledge about the effects of fatty acids (FAs) on connexin-based channels, as well as discuss the limited information about the impact FAs may have on pannexins (Panxs). FAs regulate diverse cellular functions, some of which are explained by changes in the activity of channels constituted by connexins (Cxs) or Panxs, which are known to play critical roles in maintaining the functional integrity of diverse organs and tissues. Cxs are transmembrane proteins that oligomerize into hexamers to form hemichannels (HCs), which in turn can assemble into dodecamers to form gap junction channels (GJCs). While GJCs communicate the cytoplasm of contacting cells, HCs serve as pathways for the exchange of ions and small molecules between the intra and extracellular milieu. Panxs, as well as Cx HCs, form channels at the plasma membrane that enable the interchange of molecules between the intra and extracellular spaces. Both Cx- and Panx-based channels are controlled by several post-translational modifications. However, the mechanism of action of FAs on these channels has not been described in detail. It has been shown however that FAs frequently decrease GJC-mediated cell-cell communication. The opposite effect also has been described for HC or Panx-dependent intercellular communication, where, the acute FA effect can be reversed upon washout. Additionally, changes in GJCs mediated by FAs have been associated with post-translational modifications (e.g., phosphorylation), and seem to be directly related to chemical properties of FAs (e.g., length of carbon chain and/or degree of saturation), but this possible link remains poorly understood.

## Introduction

### Fatty acids: general characteristics

Fatty acids (FAs) are carboxylic acids classified into three groups based on the length of their aliphatic carbon tails (Layden et al., 2013). These include: (i) short (<6 carbons), (ii); medium (6–12 carbons); and (iii) long (>12 carbons) aliphatic chains (Talukdar et al., 2011; Layden et al., 2013). In addition, FAs are also classified by the number of double bonds present in their aliphatic chain: saturated fatty acids (SFAs), monounsaturated fatty acids (MUFAs) or polyunsaturated fatty acids (PUFAs) (Poudyal and Brown, 2015). In turn, PUFAs can be classified into omega-3 (ω-3) and omega-6 (ω-6), based on the location of the last double bond (Schmitz and Ecker, 2008). Despite their structural similarities, ω-3 FAs generally cause biological responses opposing to ω-6 FAs (Senkal et al., 2007). Although traditionally the interest in FAs and their effect on human health has been mainly related to cardiovascular diseases, it is now well accepted that FAs influence other diseases, including metabolic disorders such as type 2 diabetes and diseases with a significant inflammatory response (Calder, 2015).

In addition to serving as energy sources, FAs also impact the following: cell membrane properties, localization and activity of channels, receptors and transporters, and activation of intracellular signaling pathways through membrane receptors (Kim and Clapham, 1989; Schmitz and Ecker, 2008; Ichimura et al., 2009; Langelier et al., 2010; Calder, 2015). In this context, oleic acid (OA) and linoleic acid (LA) regulate the amount of GLUT4, decreasing both protein and mRNA levels in a concentration-dependent manner in skeletal muscle cells (Poletto et al., 2015). They also regulate their own metabolism, when for example palmitic acid (PA) and stearic acid (SA) stimulate intracellular lipid accumulation (Xiao et al., 2012). Additionally, FAs modulate connexin (Cx) and pannexin (Panx) functions, which play critical roles in cellular communication and the functional integrity of various organs and tissues (Sáez et al., 2005; Bedner et al., 2012). Therefore, FAs have profound effects on a myriad of cell functions, some of which could be related to the modulation of cell-cell communication mediated by Cxs and Panxs.

### Connexins and pannexins

Cxs are encoded by 20 genes in mice and 21 genes in humans (Söhl and Willecke, 2004; Bedner et al., 2012). In the endoplasmic reticulum (ER) and Golgi/Trans Golgi they assemble into hexamers, known as hemichannels (HCs) (Sáez et al., 2005; D'Hondt et al., 2009). Another possible configuration occurs when Cxs assemble into dodecamers formed by the serial docking of two HCs to form a gap junction channel (GJC), which connects the cytoplasm of contacting cells (Söhl and Willecke, 2004; Hervé and Derangeon, 2013). The role of GJCs in several cell functions depends on cell type, the Cx type expressed and the physiologic state of cells (e.g., quiescent or proliferating cells) (Jiang and Gu, 2005; Rackauskas et al., 2010; Bedner et al., 2012).

HCs form poorly selective channels (Chandrasekhar and Bera, 2012) that participate in paracrine and autocrine signaling, since they are pathways for releasing signaling molecules such as: ATP, PGE_2_ and glutamate (Sáez et al., 2005). GJCs on the other hand enable the interchange of metabolites and second messengers between contacting cells. Hence, both GJCs and HCs are fundamental for several relevant functions under physiological and pathophysiological conditions (for further details see Sáez et al., 2003; Chandrasekhar and Bera, 2012; Retamal et al., 2015).

Panxs as Cxs present four transmembrane domains, two extracellular loops, one intracellular loop, and both N- and C-termini facing the cytosol. Both human and mouse genomes contain the following three Panx-encoding genes: Panx1, Panx2, and Panx3 (Baranova et al., 2004). Panx1 and Panx3 are composed of 6 subunits, whereas Panx2 is composed of 8 subunits (Ambrosi et al., 2010; Boassa et al., 2015). All Panxs form channels at the plasma membrane, but Panx3 also forms channels at the ER, where it seems to control calcium flux in this organelle (Ishikawa et al., 2011). As mentioned previously, Cxs and Panxs share a similar topology at the plasma membrane, and share certain functional properties. Thus, under normal conditions, both Cx HCs and Panx channels have very low open probabilities (Quist et al., 2000; Contreras et al., 2003), which can be increased (Chandrasekhar and Bera, 2012), for example, by raising the intracellular Ca^2+^ concentration (Locovei et al., 2006; De Vuyst et al., 2009). Another similarity between Cx HCs and Panx1 channels is that they are also permeable to ions and small signaling molecules (Panchin, 2005; Locovei et al., 2006).

## Regulation of gap junction channels by fatty acids

Cxs are regulated by post-translational modifications, such as phosphorylation and S-nitrosylation (Retamal et al., 2006; Johnstone et al., 2012; D'Hondt et al., 2013). The regulation by FAs or their conjugated version however has received little attention. The first reports showed that OA (18:1), arachidonic acid (AA, 20:4) and docosahexaenoic acid (DHA, 22:6) are powerful inhibitors of GJCs in the heart, vascular smooth muscle cells, and liver epithelial cell lines (Hirschi et al., 1993; Hii et al., 1995). This inhibition effect was reversed upon washing the cells with a FA-free solution (Hirschi et al., 1993; Hii et al., 1995), suggesting that this response might be mediated either by direct FA interaction with the GJC or by activation of a membrane receptor. Additionally, the effect of OA was concentration-dependent, with a greater inhibitory effect at low OA concentrations (Hirschi et al., 1993). In cells derived from rat lacrimal glands, an inhibitory effect of PUFAs or SFAs over GJCs was also reported (Giaume et al., 1989). Thus, AA, LA (18:2) or lauric acid (12:0) induces GJC closure, an effect that is not prevented by inhibitors of AA metabolism (Giaume et al., 1989), excluding cyclooxygenase products as possible mediators (Schmilinsky-Fluri et al., 1997). Moreover, AA decreases junctional conductance in neonatal rat heart cells (Fluri et al., 1990). This effect is specific to the degree of AA saturation, because arachidic acid (a structural saturated analog of AA) showed no effect on GJC conductance (Fluri et al., 1990). AA-induced cell-cell uncoupling was also shown to be reversible and could be mediated by direct action on Cx proteins (Fluri et al., 1990). Alternatively, AA or other PUFAs, due to their amphipathic character, could interfere with GJC conductance by disturbing the lipid-protein interface (Schmilinsky-Fluri et al., 1997). The possibility that the FA-induced GJC-inhibition could be a consequence of changes in distribution or expression of Cxs has not been tested.

Another characteristic of FAs that could be significant in the regulation of GJCs is the length of their carbon chains. Research has proven that FAs of different lengths have unique chemical properties (Layden et al., 2013). In fact, perfluorinated FAs (PFFAs), which are FA analogs (Kudo et al., 2011), were shown to inhibit GJCs in a concentration-dependent manner in a liver epithelial cell line due to their aliphatic chains ranging from 7 to 10 carbons. PFFAs, on the other hand, with 2–5 or 16–18 carbon chain lengths had no effect on GJCs (Upham et al., 1998, 2009). Additionally, the inhibition by PFFAs was observed after 5 min of incubation, and involved an ERK-dependent pathway (Upham et al., 2009). Moreover, the short-term inhibition induced by PFFAs in GJCs was lost after washing the cells, which suggests the involvement of an extracellular component. In this case, Upham et al. (2009) also reported on prevention with the presence of different protein kinase inhibitors. It is thus possible that PFFAs activate membrane receptors, which in turn activate signaling proteins such as protein kinases (Upham et al., 2009). It should also be considered that PFFAs with a carbon chain between 6 and 9 carbons increase the liver FA profile after 5 days of treatment, with specific increases in palmitoleic acid (PO, 16:1), OA (18:1) and eicosatrienoic acid (20:3) content (Kudo et al., 2011). Consequently, treatment under different exposure times may also induce different effects (e.g., acute vs. chronic treatments).

Inhibition of GJCs induced after 1 h treatment with LA was reversible in a rat liver epithelial cell line (Hayashi et al., 1997), and was probably mediated by an intracellular signaling pathway (such as PKA). In contrast, cell response to long-term treatments with LA (e.g., 6 days) was not recovered after washing out the extracellular solution. This may suggest that regulation could involve post-translational modifications at least for Cx43, as suggested by Hayashi et al. (1997). PUFAs also regulated GJC activity in a transfected cell line. For example, AA inhibited GJCs in Cx36 transfected HeLa cells, and GJC activity was restored after washout (Marandykina et al., 2013).

On the other hand, different FAs might act as activators, since they increase both GJC and HC activity. For example, in human endothelial cells, a reduction of Cx43 GJC coupling induced by hypoxia/reoxygenation was observed, but this effect was not detected in cells pretreated (for 2 days) with 10 μM EPA (20:5), a ω-3 PUFAs (Zhang et al., 1999, 2002). Accordingly, in rat astrocytes supplemented for 10 days with DHA, an increase in gap junctional communication was recorded (Champeil-Potokar et al., 2006). Also, the ω-6 gamma-linolenic acid (GLA, 18:3) increased Cx43 GJC activity in human vascular endothelial cells (Jiang et al., 1997).

Although it is not easy to characterize the effect of PUFAs on GJC activity, the effect of AA has been consistently associated to the same response: inhibition of GJC activity (Giaume et al., 1989; Fluri et al., 1990; Hii et al., 1995; Marandykina et al., 2013). But curiously, this type of response is absent in *Xenopus* oocytes, where AA does not affect GJCs formed by Cx46 (Retamal et al., 2011). This suggests that the signaling pathway associated to the AA response is missing in *Xenopus* oocytes. This situation could be a consequence of (1) different lipid compositions of the cell membrane (e.g., levels and/or distribution of cholesterol) or (2) absence of either specific extra- or intracellular signaling molecules (e.g., membrane receptors or protein kinases).

## Connexin modifications induced by fatty acids

PUFAs are known to induce changes in the expression, distribution, and post-translational modifications of Cx proteins, which have been found to be correlated with changes in GJC activity. In particular, GLA decreases Cx43 tyrosine phosphorylation in human vascular endothelial cells (Jiang et al., 1997), while OA enhances the phosphorylate state at Cx43-S368 in rat cardiomyocytes (Huang et al., 2004). This post-translational modification has been associated with the disassembly and/or closure of Cx43-GJCs (Huang et al., 2004; Solan and Lampe, 2014). Accordingly, DHA alone or with EPA increases Cx43 phosphorylation in rat astrocytes and vascular endothelial cells (Champeil-Potokar et al., 2006; Dlugosova et al., 2009; Radosinska et al., 2013). The participation of different protein kinases, such as PKA, PKC-epsilon, PI3K, AKT, Src, or MEK1/2, has been observed in this type of Cx regulation (Popp et al., 2002; De Vuyst et al., 2007; Figueroa et al., 2013; Radosinska et al., 2013). All these *in vitro* data corroborate what happens *in vivo*. Thus, under physiological conditions, the content of astrocytic Cx43 has been directly associated with DHA concentration in rat brain (Maximin et al., 2015).

Regulation of Cxs by other mechanisms has also been reported. In rat models of hypertensive and hypertriglyceremic diseases, treatment with DHA + EPA (between 3 weeks and 2 moths) restores the expression levels and distribution of Cx43 at the cell membrane (Fischer et al., 2008; Mitasíková et al., 2008; Dlugosova et al., 2009; Bacova et al., 2010). In rat neural stem cells, Cx43 increases only in lipid rafts (with no changes in total Cx43) after 3 days of treatment with DHA, suggesting that this effect was only due to a redistribution of Cx43 at the cell membrane (Langelier et al., 2010). This Cx43 response within lipid rafts is not so unexpected, because these membrane domains (which are cholesterol-rich) are associated with trafficking of membrane proteins (McIntosh et al., 2003; Sánchez et al., 2010), including some Cx types (e.g., Cx32, Cx36, Cx43, and Cx46), which are preferentially located inside the lipid rafts. Interestingly, other Cxs (e.g., Cx26 and Cx50) are preferentially found outside these membrane domains (Schubert et al., 2002; Defamie and Mesnil, 2012). Cholesterol levels seem to play an important role in the regulation of Cx43, as seen in a cell line derived from rat cardiomyocytes (H9c2 cells). This is because cholesterol enrichment reduced dye transfer through Cx43 GJCs, due to activation of a PKC-dependent signaling pathway that induces Cx43 phosphorylation at S368 (Palatinus et al., 2011; Zou et al., 2014). A second residue may also be involved, because phosphorylation on S365 inhibits phosphorylation of the amino acid residue S368 (Solan and Lampe, 2014). Moreover, the assembly of GJCs and their activity are regulated by the lipid composition of the cell membrane (Defamie and Mesnil, 2012). Interestingly, differences in lipid composition of the plasma membrane could explain the different cell- or Cx-dependent responses. For instance, in two different human hepatoma cell lines (HepG2 and SMMC-7721) the increase in GJC activity induced by retinoic acid is associated to an increase in the amount of Cx43 (HepG2 cells) or Cx32 (SMMC-7721 cells) (Yang et al., 2014).

## Hemichannel activity and fatty acids

The effects of FAs on Cx HC activity were only described in the last decade. Electrophysiological experiments have shown that pro-inflammatory PUFAs induce a biphasic effect in Cx46 HCs expressed in *Xenopus oocytes*. Thus, LA increases Cx46 HC currents at a low concentration (0.1 μM), and decreases HC currents at a high concentration (100 μM). The maximum inhibitory effect was completed in 2 min, and the inhibition was reversible after washout (Retamal et al., 2011). This biphasic response was also suggested for Cx43, because 11,12-epoxyeicosatrienoic acid, which is a metabolic derivative of AA (Spector et al., 2004), transiently increased cell coupling followed by a sustained uncoupling in human endothelial cells (Popp et al., 2002).

LA was also shown to increase HC activity in HeLa cells transfected with Cx26, Cx32, Cx43, or Cx45 within a few minutes of esposure (Figueroa et al., 2013). LA also increased Cx43 HC activity in a cell line derived from human gastric epithelial cells (Puebla et al., 2016). In this case, the effect was mediated by the activation of GPR40 (a membrane receptor) and intracellular AKT-dependent signaling pathway (Puebla et al., 2016). The GPR40 receptor belongs to a group of G-protein-coupled receptors (GPCRs) that include 5 other membrane receptors, which are also activated by FAs (Talukdar et al., 2011). These receptors are proposed to play critical roles in various physiological and pathophysiological conditions (Miyauchi et al., 2010; Talukdar et al., 2011).

To date, the information on Panx regulation by FAs is limited. It has been shown that Panx1 and Panx3 are regulated by certain FAs. In human and rat liver cell lines, acute treatment with SFAs such as PA (16:0) and SA (18:0) increases Panx1 channel activity. This response contributes to ATP release, which finally induces apoptosis (Xiao et al., 2012). Apparently, the regulation of Panx1 channel activity by FAs would depend on the degree of unsaturation of the FA in question. For example, the monounsaturated versions of PA and SA [i.e., PO (16:1) and OA (18:1)] do not affect Panx1 channel activity. Conversely, a PUFA as AA (20:4) reduces the macroscopic membrane current of Panx1 channels expressed in *Xenopus* oocytes, and reduces the release of ATP (Xiao et al., 2012; Samuels et al., 2013). With regard to Panx3, treatment of L6 myotubes with palmitate, but not palmitolate, was observed to promote the release of a macrophage chemoattracting agent likely to be ATP-released through Panx3 channels, since it was abrogated after silencing Panx3 (Pillon et al., 2014).

## Conclusion

**Figure 1:**
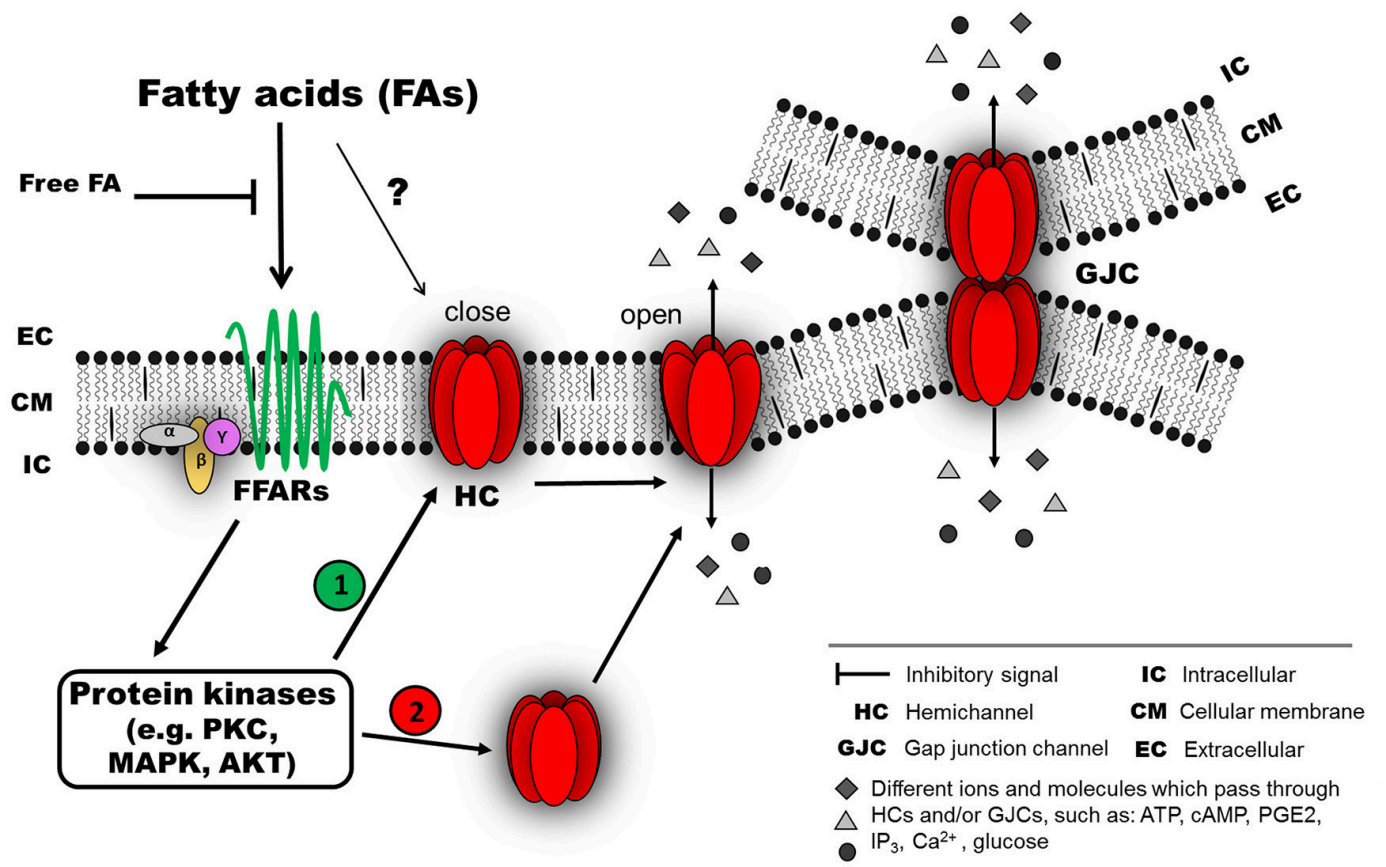
**Possible mechanism of connexin responses associated with acute exposure to fatty acids (FAs)**. FAs induce connexin (Cx) responses by interacting with membrane receptors, such as members of the G-protein coupled receptors (GPCRs), for example, GPR40 (free fatty acid receptor 1, FFAR1) or GPR120 (FFAR4), which are activated by medium- and long-chain FAs (Ichimura et al., 2014). The activation of these FFARs could involve an intracellular signaling pathway associated with different protein kinases (e.g., PKC, MAPK or AKT). Then, the activated kinase could induce at least two different effects: (1) modification of the open probability of Cx hemichannels (HCs) and/or gap junction channels (GJC), and/or (2) modification of Cx abundance in the cellular membrane (as HCs or GJCs) by changing the relative amount and distribution of intracellular Cx. In this model, the intracellular signaling associated with FFAR activation is lost after a wash out with a FA-free solution. A direct interaction between FAs and Cxs cannot be ruled out (?).

**Figure 2:**
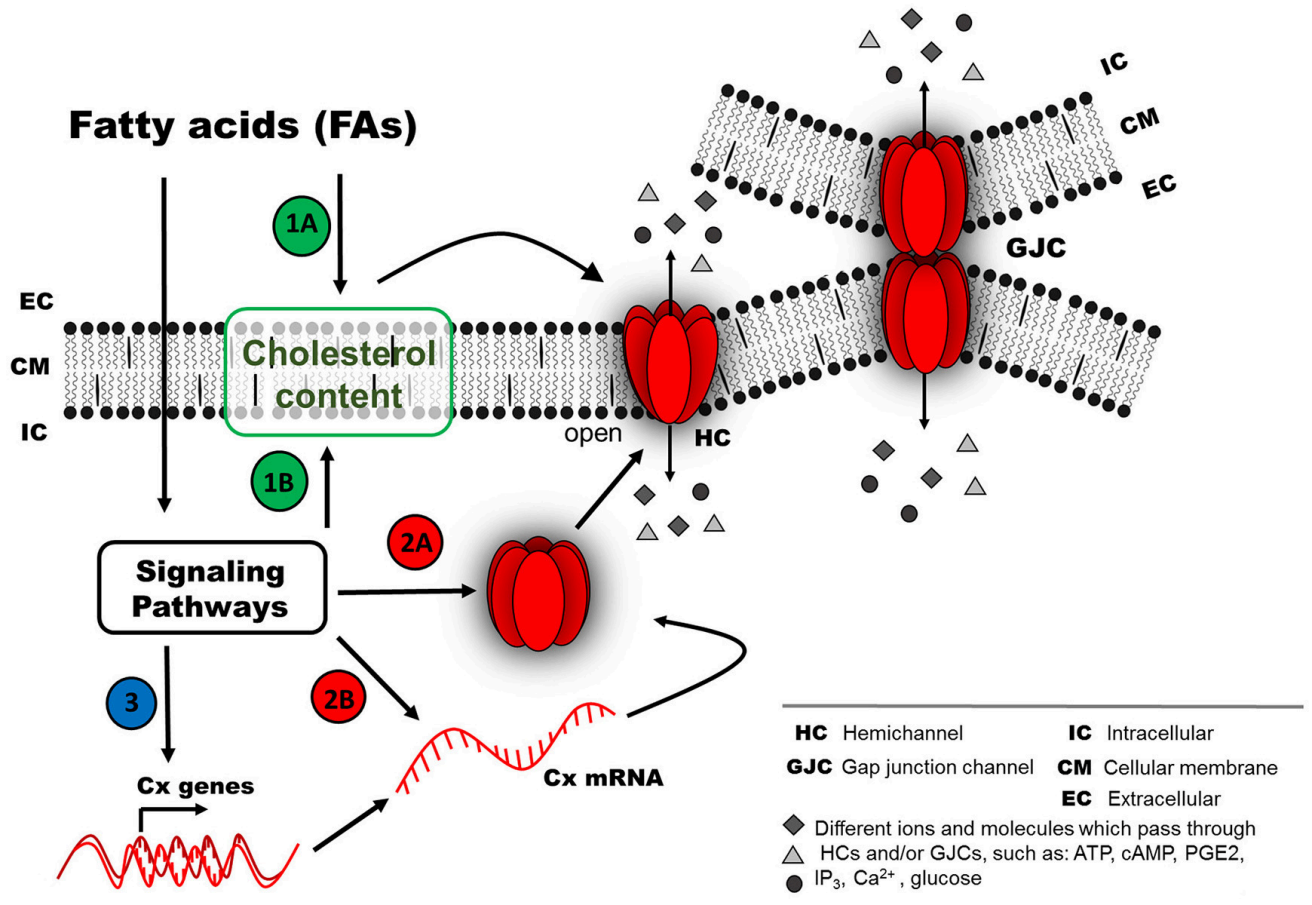
**Possible mechanism of connexin responses associated with chronic exposure to fatty acids (FAs)**. Chronic FA exposure could induce a long-term effect (second mechanism), because the FFARs could be inactivated due to long FA exposure (e.g., internalized or down-regulated). FA effects on the activity of Cx-based channels (as HCs or GJCs) probably involve different pathways, such as (1) regulation of Cx distribution at the cellular membrane by a cholesterol-dependent mechanism, either by direct action from the extracellular space (1A) or after activation of intracellular signaling pathways (1B). (2) Signaling pathways could modify Cx protein abundance due to changes in protein degradation rate (2A) or synthesis rate (2B). (3) Another option less studied is the regulation of mRNA stability or transcriptional activity of Cx genes (e.g., epigenetic regulation).

Since the effects of brief treatments with different FAs on GJC activity are reversible upon washout, it is likely that FAs act through membrane receptors and intracellular signaling pathways. There is evidence in support of the participation of membrane receptors in the short-term effect on Cx-based channels (both GJCs and HCs). In some cases, the FA-mediated effect requires the participation of protein kinases that could be activated downstream of different GPCRs (Osmond et al., 2012; Suire et al., 2012; Liang et al., 2015), such as FA receptors (Itoh et al., 2003; Hirasawa et al., 2005). In agreement with such evidence, several protein kinases have been identified to modify Cxs (Solan and Lampe, 2014; Pogoda et al., 2016). However, a direct interaction between FAs and Cxs cannot be ruled out (Figure 1). For long-term FA treatments, the effects are not reversible upon washout, and, therefore, a second mechanism may be involved, including regulation at the level of protein synthesis and/or protein redistribution in a cholesterol-dependent way (Gibbons, 2003). Other possible mechanisms that have scarcely been explored include regulation at the level of mRNA stability, mRNA synthesis (transcriptional regulation) or epigenetic regulation (Kiec-Wilk et al., 2011; Salat-Canela et al., 2015). Related to the latter, an increase in methylation of the Cx43 gene induced by AA in endothelial progenitor cells have been described (Kiec-Wilk et al., 2011) (Figure 2).

Cx regulation is critical for several cell functions and a large number of diseases can be attributed to changes in expression, function and/or properties of these proteins (Hills et al., 2015), it may be possible to suggest that the effect of FAs upon Cx-based channels can have an important impact in translational research. Thus, the uses of FAs that suppress HC activity in diseases where Cx HC activity is upregulated (e.g., ischemia reperfusion) could have important treatment benefits. Additionally, certain FAs could provide significant advantages in diabetic nephropathy, for instance, where they may restore the loss of GJC-mediated cell-cell communication within the nephron (Hills et al., 2015). Other examples where FAs may be used in addition to current therapies, and in which Cxs play important roles are lens cataracts (Beyer and Berthoud, 2014) and cancer, where Cxs have different (and controversial) types of participation (Aasen et al., 2016). Another advantage in the use of FAs for certain diseases is that many of them are harmless to humans. Alternatively, it may also be possible to develop modified FAs with higher specificities for Cx docking.

## Author contributions

All authors listed have made substantial, direct and intellectual contributions to the work, and approved it for publication.

### Conflict of interest statement

The authors declare that the research was conducted in the absence of any commercial or financial relationships that could be construed as a potential conflict of interest.
